# A Molecular Electron Density Theory Study of the Chemical Reactivity of Cis- and Trans-Resveratrol

**DOI:** 10.3390/molecules21121650

**Published:** 2016-12-01

**Authors:** Juan Frau, Francisco Muñoz, Daniel Glossman-Mitnik

**Affiliations:** 1 Universitat de les Illes Balears, Departament de Química, 07122 Palma de Mallorca, Spain; juan.frau@uib.es (J.F.); paco.munoz@uib.es (F.M.); 2 Laboratorio Virtual NANOCOSMOS, Centro de Investigación en Materiales Avanzados, Departamento de Medio Ambiente y Energía, Chihuahua, Chih 31136, Mexico

**Keywords:** computational chemistry, molecular modeling, cis-Resveratrol, trans-Resveratrol, conceptual DFT, molecular electron density theory

## Abstract

The chemical reactivity of resveratrol isomers with the potential to play a role as inhibitors of the nonenzymatic glycation of amino acids and proteins, both acting as antioxidants and as chelating agents for metallic ions such as Cu, Al and Fe, have been studied by resorting to the latest family of Minnesota density functionals. The chemical reactivity descriptors have been calculated through Molecular Electron Density Theory encompassing Conceptual DFT. The active sites for nucleophilic and electrophilic attacks have been chosen by relating them to the Fukui function indices, the dual descriptor $f^{\left(2\right)}\left(r\right)$ and the electrophilic and nucleophilic Parr functions. The validity of “Koopmans’ theorem in DFT” has been assessed by means of a comparison between the descriptors calculated through vertical energy values and those arising from the HOMO and LUMO values.

## 1. Introduction

Following the pioneering work of Parr and others [1], a useful number of concepts have been derived from the analysis of the density of any molecular system through Density Functional Theory (DFT). These concepts that allow a researcher to make qualitative predictions about the chemical reactivity of a given system can also be quantified and are collectively known as Conceptual DFT Descriptors.

In order to obtain quantitative values of the Conceptual DFT Descriptors, it is necessary to resort to the Kohn–Sham theory through calculations of the molecular density, the energy of the system, and the orbital energies—in particular, those related to the frontier orbitals, known as HOMO and LUMO [2,3,4,5,6,7].

More recently, Luis R. Domingo [8] proposed a new theory for the study of the reactivity in Organic Chemistry, which he named Molecular Electron Density Theory (MEDT). In this new theory, the capability for changes in electron density is responsible for the molecular reactivity [8], while the electron density distribution at the ground state is responsible for physical and chemical molecular properties.

Within MEDT, which encompasses Conceptual DFT, several new concepts and reactivity descriptors have been defined as the Global Electron Density Transfer (GEDT), the Nucleophilicity N index, and local condensed descriptors like the electrophilic $P_{k}^{-}$ and nucleophilic $P_{k}^{+}$ Parr functions [9]. A usual way to proceed implies as a first step the choice of a model chemistry for the study of the molecular system or chemical reaction of interest. A model chemistry is a combination of a density functional, a basis set, and an implicit solvent model that one considers, which can be adequate for the problem under study. There is a plethora of information in the literature about how to choose this model chemistry, and one generally follows the experience of previous researchers and his/her own work.

Although the foundations of DFT have established that a universal density functional must exist, and that all of the properties of the system can be obtained through calculations with this functional, in practice, one needs to resort to some of the approximate density functionals that have been developed during the last thirty years. Due to the fact that these are approximate functionals (that is, not universal functionals), many of them are good for predicting some properties and others are good for other properties. Sometimes, you can find density functionals that are excellent for describing the properties of a given molecular system with a particular functional group, but it is necessary to resort to other density functionals for a different functional group that you want to include in the molecular system under study.

When one is dealing with the study of the chemical reactivity, that is, a process that involves the transference of electrons, it is usual to perform calculations not only of the ground state, but also for open systems like the radical cation and radical anion. These systems are often difficult to converge while giving trustworthy results, especially if diffuse functions must be included in the basis set [2,3,4,5,6,7]. For this reason, it is convenient to have a method that can give all information that one needs directly from the results of the calculation of the ground state of the molecular system under study. In particular, one may want to obtain the ionization potential (I) and electron affinity (A) of the system avoiding the calculation of the radicals anion and cation. Indeed, the link for this is given by the so-called Koopmans’ theorem [4,5,6,7], which states that within Hartree–Fock (HF) theory, the I can be approximated by subtracting the energy of the HOMO, that is, I = −$\epsilon_{H}$. By extension, it is considered that the A can be approximated by subtracting the energy of the LUMO, that is, A = −$\epsilon_{L}$.

It has been mentioned recently [10] that an exact physical meaning can be assigned to the Kohn–Sham (KS) HOMO using the “KS analog of Koopmans’ theorem in Hartree–Fock theory”, which states that for the exact theory, the KS HOMO is equal to and opposite of the ionization potential, $\epsilon_{H}$ = −I [11,12,13,14]. Due to the aforementioned problem of discontinuity, a similar Koopmans’ theorem that relates the LUMO energy to the electron affinity does not exist. Thus, it has been proposed, in order to circumvent the problem, to consider that the I of the N+1 electron system (the anion) is the same as that the A of N electron system [10]. By considering range-separated hybrids (RSH) functionals [15,16,17], Kronik et al. [10] showed that with a judicious choice of the range-separation parameter *γ*, the validity of Koopmans’ theorem could be enforced. For example, Lima et al. [18] have recently presented an improved description of the optical properties of carotenoids by tuning some RSH corrected density functionals.

This means that the goodness of a given density functional can be estimated by checking how well it follows “Koopmans’ theorem in DFT”, which makes it behave closer to the exact density functional, and this will be crucial for a good calculation of the Conceptual DFT descriptors that predict and explain the chemical reactivity of molecular systems. However, the *γ* tuning procedure for the RSH density functionals is system dependent, and this implies that different density functionals are going to be used for the calculation of the descriptors for the different molecular systems. Thus, it will be interesting to study other RSH density functionals where the *γ* parameter is fixed by construction, although other parameters have been fitted to reproduce some molecular properties. In particular, we are going to consider several density functionals that have been tested against a large number of databases in chemistry and physics [19].

**Figure 1:**
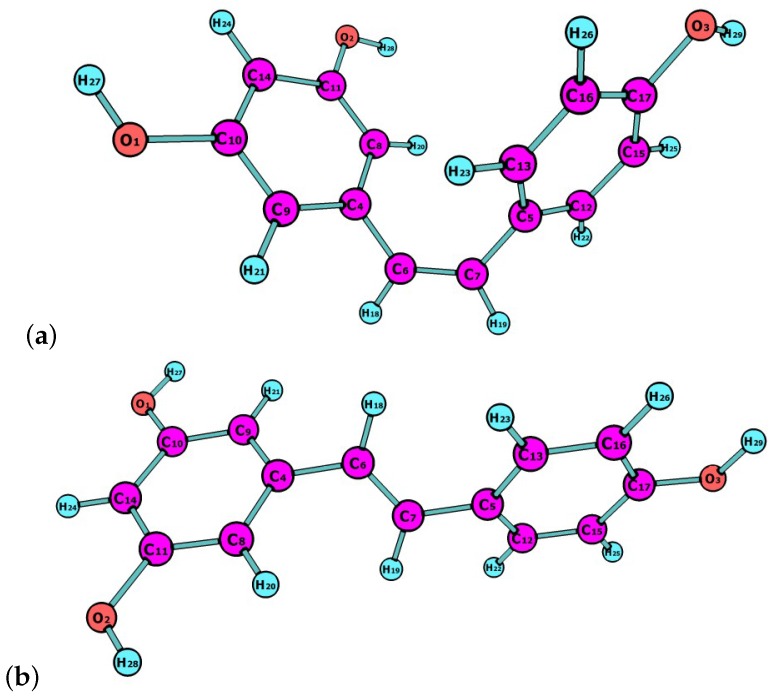
Molecular structures of (**a**) cis-resveratrol; and (**b**) trans-resveratrol.

Resveratrol is a natural compound that is synthesized by plants in response to adverse conditions, such as environmental stress or pathogenic attacks. Resveratrol exists as ltrans and cis isomers, both found in wines and other plant fruits with different concentrations depending on weather conditions [20]. They could be helpful in preventing the nonenzymatic glycation of amino acids and proteins by acting as antioxidants and through the complexation of metals, like Cu, Al and Fe [21,22,23]. In this work, the latest Minnesota density functionals will be considered for the study of chemical reactivity descriptors of both isomers, of which molecular structures are shown in Figure 1.

## 2. Theoretical Background

The chemical potential *μ* has been defined as [24,25]:

(1) $$\mu ={\left(\frac{\partial E}{\partial N}\right)}_{v(\vec{r})}=-\chi ,$$

where *χ* is the global electronegativity, and the global hardness *η* can be expressed as:

(2) $$\eta ={\left(\frac{\partial^{2}E}{\partial N^{2}}\right)}_{v(\vec{r})}.$$

The expressions above can be written as [4,5,6,7]:

(3) $$\mu =-\frac{1}{2}\left(I+A\right)\approx \frac{1}{2}\left(\epsilon_{L}+\epsilon_{H}\right)=\chi_{K},$$

(4) $$\eta =\left(I-A\right)\approx \left(\epsilon_{L}-\epsilon_{H}\right)=\eta_{K},$$

where $\epsilon_{H}$ and $\epsilon_{L}$ are the energies of the highest occupied and the lowest unoccupied molecular orbitals, HOMO and LUMO, respectively. The use of the energies of frontier molecular orbitals as an approximation to obtain *I* and *A* is supported by “Koopmans in DFT” (KID) procedure.

The electrophilicity index *ω* is expressed as:

(5) $$\omega =\frac{\mu^{2}}{2\eta }=\frac{{\left(I+A\right)}^{2}}{4(I-A)}\approx \frac{{\left(\epsilon_{L}+\epsilon_{H}\right)}^{2}}{4(\epsilon_{L}-\epsilon_{H})}=\omega_{K},$$

while the electrodonating ($\omega^{-}$) and electroaccepting ($\omega^{+}$) powers have been defined as [26]:

(6) $$\omega^{-}=\frac{{\left(3I+A\right)}^{2}}{16(I-A)}\approx \frac{{\left(3\epsilon_{H}+\epsilon_{L}\right)}^{2}}{16\eta_{K}}=\omega_{K}^{-},$$

and

(7) $$\omega^{+}=\frac{{\left(I+3A\right)}^{2}}{16(I-A)}\approx \frac{{\left(\epsilon_{H}+3\epsilon_{L}\right)}^{2}}{16\eta_{K}}=\omega_{K}^{+}.$$

Then, a system with a large value of $\omega^{+}$ will have better capability of accepting charge, and a molecule with a small $\omega^{-}$ value will be a better electron donor. Chattaraj et al. [27] have proposed the following definition of net electrophilicity:

(8) $$\Delta \omega^{\pm }=\omega^{+}-\left(-\omega^{-}\right)=\omega^{+}+\omega^{-}\approx \omega_{K}^{+}-\left(-\omega_{K}^{-}\right)=\omega_{K}^{+}+\omega_{K}^{-}=\Delta \omega_{K}^{\pm }.$$

The Fukui function f(r) is defined in terms of the derivative of ρ(r) with respect to *N* [1,25]:

(9) $$f\left(r\right)={\left(\frac{\partial \;\rho (r)}{\partial \;N}\right)}_{\upsilon (r)}$$

and reflects the ability of a molecular site within a molecule to accept or donate electrons. The Fukui function f(r) can be condensed to reflect the site for a nucleophilic attack $f^{+}\left(r\right)$ or an electrophilic attack $f^{-}\left(r\right)$ [24].

Morell et al. [28,29,30,31,32,33,34] have proposed a local reactivity descriptor (LRD), which is called the dual descriptor (DD) $f^{\left(2\right)}\left(r\right)\equiv \Delta f\left(r\right)$. The condensation to atoms of the dual descriptor leads to $f_{k}^{\left(2\right)}$, so that when $f_{k}^{\left(2\right)}>0$, the site represented by atom *k* will be prone to a nucleophilic attack, while, for the case of $f_{k}^{\left(2\right)}<0$, the process will be driven by an electrophilic attack over atom *k*.

In 2014, Domingo proposed the Parr functions P(r) [35,36] that are given by the following equations:

(10) $$P^{-}\left(r\right)=\rho_{s}^{rc}\left(r\right)$$

for electrophilic attacks, and

(11) $$P^{+}\left(r\right)=\rho_{s}^{ra}\left(r\right)$$

for nucleophilic attacks, which are related to the atomic spin density (ASD) at the r atom of the radical cation or anion of a given molecule, respectively. The ASD over each atom of the radical cation and radical anion of the molecule gives the local nucleophilic $P_{k}^{-}$ and electrophilic $P_{k}^{+}$ Parr functions of the neutral molecule [9].

## 3. Settings and Computational Methods

The Gaussian 09 [37] series of programs with density functional methods as implemented in the computational package were used for the calculation of the optimized geometries. The basis set used in this work was Def2SVP for geometry optimization and frequencies, while Def2TZVP was considered for the calculation of the electronic properties [38,39].

Several density functionals from the latest Minnesota family were considered: M11, which is a is a range-separated hybrid meta-GGA [40], M11L, which is a dual-range local meta-GGA [41], MN12L, which is a nonseparable local meta-NGA [42], MN12SX, which is a range-separated hybrid nonseparable meta-NGA [43], N12, which is a nonseparable gradient approximation [44], N12SX, which is a range-separated hybrid nonseparable gradient approximation [43], SOGGA11, which is a GGA density functional [45] and SOGGA11X, which is a hybrid GGA density functional [46]. In these functionals, GGA stands for generalized gradient approximation and NGA stands for nonseparable gradient approximation. All the calculations were performed in the presence of water as a solvent by doing IEF-PCM computations according to the SMD solvation model [47].

## 4. Results and Discussion

The molecular structures of cis- and trans-resveratrol were pre-optimized by starting with the readily available MOL structures, and finding the most stable conformer by means of the Avogadro 1.2.0 program (Open Molecules, Pittsburgh, PA, USA) [48,49]. The structures of the resulting conformers were then reoptimized with the M11, M11L, MN12L , MN2SX, N12, N12SX, SOGGA11 and SOGGA11X density functionals in connection with the Def2SVP basis set and the SMD solvation model, using water as a solvent.

For the purpose of this study, it is worth calculating the electronegativity *χ*, the global hardness *η* and the global electrophilicity *ω* for the studied systems using both approximations in order to verify the quality of the procedures. Additionally, we will include in the calculations the electrodonating ($\omega^{-}$) and electroaccepting ($\omega^{+}$) powers as well as the net electrophilicity $\Delta \omega^{\pm }$ for further verifications.

The HOMO and LUMO orbital energies (in eV), ionization potentials I and electron affinities A (in eV), and global electronegativity *χ*, total hardness *η*, global electrophilicity *ω*, electrodonating power, ($\omega^{-}$), electroaccepting power ($\omega^{+}$), and net electrophilicity $\Delta \omega^{\pm }$ of the cis- and trans-resveratrol molecules calculated with the M11, M11L, MN12L, MN12SX, N12, N12SX, SOGGA11, and SOGGA11X density functionals and the Def2TZVP basis set using water as a solvent simulated with the SMD parametrization of the IEF-PCM model are presented in Table 1 and Table 2, respectively. The upper part of the tables shows the results derived assuming the validity of Koopmans’ theorem in DFT (hence the subscript K) and the lower part shows the results derived from the calculated vertical I and A.

With the object of analyzing our results in order to verify the fulfillment of “Koopmans’ theorem in DFT”, we have designed several descriptors that relate the results obtained through the HOMO and LUMO calculations with those obtained by means of the vertical I and A with a ΔSCF procedure. However, it must be stressed that it is not our intention to perform a gap-fitting by minimizing a descriptor by choosing optimal range-separation parameter *γ*, but to check if the density functionals considered in this study, in which some of them contain a fixed range-separation parameter *γ*, obey “Koopmans’ theorem in DFT”. As a matter fact, there is no range-separation parameter *γ* in our designed descriptors. Moreover, we have considered A by subtracting the energy of the LUMO of the neutral system instead of considering A by subtracting the energy of the HOMO of the N+1 electron system, as it was in the mentioned works [10,18].

The first three descriptors are related to the simplest fulfillment of Koopmans’ theorem by relating $\epsilon_{H}$ with -I, $\epsilon_{L}$ with -A, and the behavior of them in the description of the band gap:

(12) $$J_{I}=\left|\epsilon_{H}+E_{gs}\left(N-1\right)-E_{gs}\left(N\right)\right|,$$

(13) $$J_{A}=\left|\epsilon_{L}+E_{gs}\left(N\right)-E_{gs}\left(N+1\right)\right|,$$

(14) $$J_{Gap}=\sqrt{{J_{I}}^{2}+{J_{A}}^{2}}.$$

Next, we consider four other descriptors that analyze how much the studied density functionals are useful for the prediction of the electronegativity *χ*, the global hardness *η* and the global electrophilicity *ω*, and for a combination of these Conceptual DFT descriptors, just considering the energies of the HOMO and LUMO or the vertical I and A:

(15) $$J_{\chi }=\left|\chi -\chi_{K}\right|,$$

(16) $$J_{\eta }=\left|\eta -\eta_{K}\right|,$$

(17) $$J_{\omega }=\left|\omega -\omega_{K}\right|,$$

(18) $$J_{D1}=\sqrt{J_{\chi }^{2}+J_{\eta }^{2}+J_{\omega }^{2}},$$

where D1 stands for the first group of Conceptual DFT descriptors.

Finally, we designed other four descriptors to verify the goodness of the studied density functionals for the prediction of the electroaccepting power $\omega^{+}$, the electrodonating power $\omega^{-}$, the net electrophilicity $\Delta \omega^{\pm }$ , and for a combination of these Conceptual DFT descriptors, just considering the energies of the HOMO and LUMO or the vertical I and A:

(19) $$J_{\omega^{+}}=\left|\omega^{+}-\omega_{K}^{+}\right|,$$

(20) $$J_{\omega^{-}}=\left|\omega^{-}-\omega_{K}^{-}\right|,$$

(21) $$J_{\Delta \omega^{\pm }}=\left|\Delta \omega^{\pm }-\Delta \omega_{K}^{\pm }\right|,$$

(22) $$J_{D2}=\sqrt{J_{\omega^{-}}^{2}+J_{\omega^{+}}^{2}+J_{\Delta \omega^{\pm }}^{2}},$$

where D2 stands for the first group of Conceptual DFT descriptors.

The results of the calculations of $J_{I}$, $J_{A}$, $J_{Gap}$, $J_{\chi }$, $J_{\eta }$, $J_{\omega }$, $J_{D1}$, $J_{\omega^{+}}$, $J_{\omega^{-}}$, $J_{\Delta \omega^{\pm }}$ and $J_{D2}$ for the cis- and trans-resveratrol molecules are displayed in Table 3 and Table 4, respectively.

As can be seen from Table 1 and Table 2, and the results presented in Table 3 and Table 4, “Koopman’s theorem in DFT” holds with great accuracy for the MN12SX and N12SX density functionals, which are range-separated hybrid meta-NGA and hybrid NGA density functionals, respectively. Indeed, the values of $J_{I}$, $J_{A}$ and $J_{Gap}$ are not exactly zero. However, their values can be favorably compared with the results presented for these quantities in the work of Lima et al. [18], where the minima has been obtained by choosing a parameter that enforces that behavior.

It is interesting to see that the same density functionals also fulfill “Koopmans’ theorem in DFT” for the other descriptors, namely $J_{\chi }$, $J_{\eta }$, $J_{\omega }$, and $J_{D1}$, as well as for $J_{\omega^{-}}$, $J_{\omega^{+}}$, $J_{\Delta \omega^{\pm }}$, and $J_{D2}$ . These results are very important because they show that it is not enough to rely only on $J_{I}$, $J_{A}$ and $J_{Gap}$. For example, if we consider only $J_{\chi }$, for all of the density functionals considered, the values are very close to zero. As for the other descriptors, only the MN12SX and N12SX density functionals show this behavior. That means that the results for $J_{\chi }$ are due to a fortituous cancellation of errors.

The usual GGA (SOGGA11) and hybrid-GGA (SOGGA11X) are not good for the fulfillment of “Koopmans’ theorem in DFT”, and the same conclusion is valid for the local functionals M11L, MN12L and N12.

An important fact is that, although the range-separated hybrid NGA and hybrid meta-NGA density functionals can be useful for the calculation of the Conceptual DFT descriptors, it is not the same for the range-separated hybrid GGA (M11) density functional. An inspection of Table 1 and Table 2 shows that this is due to the fact that this functional describes inadequately the energy of the LUMO, leading to negative values of A, which are in contradiction with the ΔSCF results.

The condensed Fukui functions can also be employed to determine the reactivity of each atom in the molecule. The corresponding condensed functions are given by $f_{k}^{+}=q_{k}\left(N+1\right)-q_{k}\left(N\right)$ (for nucleophilic attack), $f_{k}^{-}=q_{k}\left(N\right)-q_{k}\left(N-1\right)$ (for electrophilic attack), and $f_{k}^{0}=[q_{k}\left(N+1\right)-q_{k}\left(N-1\right)]/2$ (for radical attack), where $q_{k}$ is the gross charge of atom *k* in the molecule. The condensed Fukui functions have been calculated using the AOMix molecular analysis program [50,51] starting from single-point energy calculations. The resulting values have been used for the determination of the condensed dual descriptor, which has been defined as $f^{\left(2\right)}{\left(r\right)}_{k}$ = $f_{k}^{+}$ − $f_{k}^{-}$ [28,29]. From the interpretation given to the Fukui function, one can note that the sign of the dual descriptor is very important to characterize the reactivity of a site within a molecule toward a nucleophilic or an electrophilic attack. That is, if $f^{\left(2\right)}{\left(r\right)}_{k}$ > 0, then the site is favored for a nucleophilic attack, whereas if $f^{\left(2\right)}{\left(r\right)}_{k}$ < 0, then the site may be favored for an electrophilic attack [28,29,52].

The condensed dual descriptor $f^{\left(2\right)}{\left(r\right)}_{k}$ over all the atoms (with the exception of the H atoms) of the cis- and trans-resveratrol molecules calculated with the M11, M11L, MN12L, MN12SX, N12, N12SX, SOGGA11 and SOGGA11X density functionals and the Def2TZVP basis set, using water as a solvent simulated with the SMD parametrization of the IEF-PCM model, are shown in Table 5 and Table 6.

The electrophilic $P_{k}^{+}$ and nucleophilic $P_{k}^{-}$ Parr functions over the atoms (excepting H atoms) of the cis- and trans-resveratrol molecules calculated with the MN12SX and N12SX density functionals and the Def2TZVP basis set using water as a solvent simulated with the SMD parametrization of the IEF-PCM model are shown in Table 7 and Table 8. We have considered ASDs coming from Hirshfeld and MBS (Minimum Basis Set) population analysis in both cases.

It can be concluded from the analysis of the results in Table 5 that all the density functionals considered in this study predict that C7 of cis-resveratrol will be the preferred site for a nucleophilic attack. At the same time, it can be said that C5 will be the site for the electrophilic attack.

If we now consider the results in Table 6 for trans-resveratrol, it is possible to see that again all the density functionals considered in this study predict that C7 of this molecule will be the preferred site for a nucleophilic attack, while C5 will be the site for the electrophilic attack.

For the case of the electrophilic $P_{k}^{+}$ and nucleophilic $P_{k}^{-}$ Parr functions, we have chosen to display only the results obtained through the use of the MN12SX and N12SX density functionals because these are the ones for which “Koopman’s theorem in DFT” holds with great accuracy. It can be seen from the values in Table 7 and Table 8 that the predicted sites for the electrophilic and nucleophilic attack for both molecules are in agreement with the results obtained through the calculation of the condensed dual descriptor $f^{\left(2\right)}{\left(r\right)}_{k}$.

## 5. Conclusions

The sites of interaction of the cis- and trans-resveratrol molecules can be predicted accurately by means of reactivity descriptors that arise from Conceptual DFT and MEDT such as the electronegativity, global hardness, global electrophilicity, electrodonating and electroaccepting powers, net electrophilicity, as well as Fukui function, condensed dual descriptor and electrophilic and nucleophilic Parr functions.

The Minnesota family of density functionals (M11, M11L, MN12L, MN12SX, N12, N12SX, SOGGA11 and SOGGA11X) have been tested for the fulfillment of “Koopmans’ theorem in DFT” by comparison of the HOMO- and LUMO-derived values with those obtained through a ΔSCF procedure. It has been shown that the range-separated hybrid meta-NGA density functional (MN12SX) and the range-separated hybrid NGA density functional (N12SX) are the best for the accomplishment of this objective. As such, they are a good alternative to those density functionals whose behavior has been tuned through a gap-fitting procedure, and a good prospect for being useful in the prediction of reactivity descriptors of molecular systems of larger size.

## Figures and Tables

**Table 1:** HOMO (Higher Occupied Molecular Orbital) and LUMO (Lower Unoccupied Molecular Orbital) energies (in eV), ionization potentials I and electron affinities A (in eV), and global electronegativity *χ*, total hardness *η*, global electrophilicity *ω*, electroaccepting power ($\omega^{+}$), and net electrophilicity $\Delta \omega^{\pm }$ of cis-resveratrol calculated with the M11, M11L, MN12L, MN12SX, N12, N12SX, SOGGA11 and SOGGA11X density functionals and the Def2TZVP basis set using water as a solvent simulated with the SMD (Solvent Model Density) parametrization of the IEF-PCM model (Integral Equation Formalism-Polarized Continuum Model). The upper part of the table shows the results derived assuming the validity of Koopmans’ theorem and the lower part shows the results derived from the calculated vertical I and A.

**Property**	**HOMO**	**LUMO**	$\chi_{K}$	$\eta_{K}$	$\omega_{K}$	$\omega_{K}^{-}$	$\omega_{K}^{+}$	$\Delta \omega_{K}^{\pm }$
M11	−8.153	0.692	3.731	8.845	0.787	3.992	0.261	4.252
M11L	−5.247	−2.088	3.667	3.159	2.129	6.289	2.622	8.910
MN12L	−5.016	−1.663	3.339	3.354	1.663	5.205	1.865	7.070
MN12SX	−5.589	−1.578	3.584	4.011	1.601	5.245	1.661	6.905
N12	−4.720	−1.882	3.301	2.838	1.920	5.667	2.366	8.034
N12SX	−5.414	−1.538	3.476	3.876	1.559	5.097	1.622	6.719
SOGGA11	−5.023	−2.203	3.613	2.820	2.314	6.611	2.998	9.609
SOGGA11X	−6.445	−0.712	3.578	5.733	1.117	4.381	0.802	5.183
**Property**	**I**	**A**	χ	η	ω	$\omega^{-}$	$\omega^{+}$	$\Delta \omega^{\pm }$
M11	6.030	1.421	3.725	4.608	1.506	5.162	1.437	6.599
M11L	5.502	1.834	3.668	3.668	1.834	5.732	2.064	7.795
MN12L	5.258	1.455	3.356	3.804	1.481	4.878	1.521	6.399
MN12SX	5.611	1.595	3.603	4.016	1.616	5.285	1.682	6.967
N12	5.025	1.562	3.294	3.462	1.566	4.996	1.703	6.699
N12SX	5.422	1.571	3.496	3.851	1.587	5.163	1.667	6.830
SOGGA11	5.323	1.887	3.605	3.436	1.891	5.799	2.194	7.993
SOGGA11X	5.688	1.512	3.600	4.176	1.552	5.165	1.565	6.730

**Table 2:** HOMO and LUMO orbital energies (in eV), ionization potentials I and electron affinities A (in eV), and global electronegativity *χ*, total hardness *η*, global electrophilicity *ω*, electroaccepting power ($\omega^{+}$), and net electrophilicity $\Delta \omega^{\pm }$ of trans-resveratrol calculated with the M11, M11L, MN12L, MN12SX, N12, N12SX, SOGGA11 and SOGGA11X density functionals and the Def2TZVP basis set using water as a solvent simulated with the SMD parametrization of the IEF-PCM model. The upper part of the table shows the results derived assuming the validity of Koopmans’ theorem and the lower part shows the results derived from the calculated vertical I and A.

**Property**	**HOMO**	**LUMO**	$\chi_{K}$	$\eta_{K}$	$\omega_{K}$	$\omega_{K}^{-}$	$\omega_{K}^{+}$	$\Delta \omega_{K}^{\pm }$
M11	−7.885	0.274	3.806	8.160	0.887	4.188	0.382	4.570
M11L	−5.116	−2.273	3.695	2.844	2.400	6.825	3.131	9.956
MN12L	−4.882	−1.848	3.365	3.034	1.867	5.605	2.240	7.846
MN12SX	−5.416	−1.819	3.617	3.597	1.819	5.672	2.054	7.726
N12	−4.614	−1.976	3.295	2.638	2.057	5.927	2.632	8.559
N12SX	−5.257	−1.733	3.495	3.524	1.733	5.435	1.940	7.374
SOGGA11	−4.919	−2.307	3.613	2.612	2.498	6.966	3.354	10.320
SOGGA11X	−6.234	0.980	3.607	5.253	1.238	4.608	1.001	5.609
**Property**	**I**	**A**	χ	η	ω	$\omega^{-}$	$\omega^{+}$	$\Delta \omega^{\pm }$
M11	5.837	1.806	3.822	4.031	1.811	5.786	1.964	7.750
M11L	5.363	2.033	3.698	3.330	2.053	6.163	2.465	8.628
MN12L	5.114	1.651	3.383	3.462	1.652	5.212	1.830	7.042
MN12SX	5.434	1.838	3.636	3.596	1.838	5.718	2.083	7.801
N12	4.911	1.665	3.288	3.246	1.666	5.178	1.890	7.068
N12SX	5.259	1.769	3.514	3.490	1.769	5.514	1.999	7.513
SOGGA11	5.220	1.993	3.606	3.227	2.015	6.035	2.428	8.463
SOGGA11X	5.491	1.771	3.631	3.719	1.772	5.593	1.962	7.554

**Table 3:** Descriptors $J_{I}$, $J_{A}$, $J_{Gap}$, $J_{\chi }$, $J_{\eta }$, $J_{\omega }$, $J_{D1}$, $J_{\omega^{+}}$, $J_{\omega^{-}}$, $J_{\Delta \omega^{\pm }}$ and $J_{D2}$ for the cis-resveratrol molecule calculated from the results of Table 1.

Descriptor	M11	M11L	MN12L	MN12SX	N12	N12SX	SOGGA11	SOGGA11X
$J_{I}$	2.124	0.255	0.242	0.021	0.305	0.008	0.300	0.757
$J_{A}$	2.113	0.254	0.208	0.017	0.320	0.033	0.316	0.801
$J_{Gap}$	2.996	0.360	0.319	0.027	0.441	0.034	0.436	1.102
$J_{\chi }$	0.005	0.001	0.017	0.019	0.008	0.020	0.008	0.022
$J_{\eta }$	4.237	0.509	0.450	0.005	0.624	0.025	0.616	1.557
$J_{\omega }$	0.719	0.295	0.182	0.015	0.353	0.028	0.423	0.435
$J_{D1}$	4.297	0.588	0.486	0.025	0.717	0.043	0.748	1.617
$J_{\omega^{-}}$	1.171	0.557	0.327	0.040	0.671	0.066	0.812	0.784
$J_{\omega^{+}}$	1.176	2.558	0.344	0.021	0.664	0.045	0.804	0.762
$J_{\Delta \omega^{\pm }}$	2.347	1.115	0.671	0.062	1.335	0.111	1.617	1.546
$J_{D2}$	2.874	1.366	0.822	0.077	1.635	0.136	1.980	1.894

**Table 4:** Descriptors $J_{I}$, $J_{A}$, $J_{Gap}$, $J_{\chi }$, $J_{\eta }$, $J_{\omega }$, $J_{D1}$, $J_{\omega^{+}}$, $J_{\omega^{-}}$, $J_{\Delta \omega^{\pm }}$ and $J_{D2}$ for the trans-resveratrol molecule calculated from the results of Table 2.

Descriptor	M11	M11L	MN12L	MN12SX	N12	N12SX	SOGGA11	SOGGA11X
$J_{I}$	2.048	0.247	0.232	0.018	0.297	0.002	0.301	0.743
$J_{A}$	2.080	0.240	0.197	0.019	0.310	0.036	0.314	0.791
$J_{Gap}$	2.919	0.344	0.304	0.026	0.430	0.036	0.435	1.085
$J_{\chi }$	0.016	0.003	0.017	0.018	0.006	0.019	0.009	0.024
$J_{\eta }$	4.128	0.487	0.429	0.000	0.608	0.034	0.615	1.534
$J_{\omega }$	0.924	0.347	0.214	0.019	0.392	0.036	0.483	0.534
$J_{D1}$	4.231	0.598	0.480	0.026	0.723	0.053	0.782	1.625
$J_{\omega^{-}}$	1.598	0.662	0.393	0.047	0.749	0.079	0.932	0.984
$J_{\omega^{+}}$	1.582	0.666	0.411	0.028	0.742	0.060	0.925	0.960
$J_{\Delta \omega^{\pm }}$	3.180	1.328	0.804	0.075	1.491	0.139	1.857	1.945
$J_{D2}$	2.874	1.366	0.822	0.077	1.635	0.136	1.980	1.894

**Table 5:** Condensed dual descriptor $f^{\left(2\right)}{\left(r\right)}_{k}$ over the atoms of the cis-resveratrol molecule calculated with the M11, M11L, MN12L, MN12SX, N12, N12SX, SOGGA11 and SOGGA11X density functionals and the Def2TZVP basis set using water as a solvent simulated with the SMD parametrization of the IEF-PCM model. The actual values have been multiplied by 100 for an easier comparison. H atoms are not shown.

Atom	M11	M11L	MN12L	MN12SX	N12	N12SX	SOGGA11	SOGGA11X
1O	−1.01	−0.96	−0.91	−0.77	−0.47	−0.56	−0.20	−0.58
2O	0.15	0.09	0.10	0.07	−0.05	0.05	−0.33	0.07
3O	−6.05	−5.34	−5.69	−5.23	−5.48	−5.28	−6.30	−5.07
4C	4.99	3.74	4.37	4.39	3.64	4.15	3.56	4.62
5C	−9.39	−8.90	−8.34	−8.76	−6.67	−7.72	−6.86	−8.67
6C	3.19	2.46	1.75	1.76	0.38	0.88	0.21	1.53
7C	8.48	10.53	9.62	8.87	7.61	7.84	7.92	7.97
8C	−0.95	−0.67	−0.47	−0.24	1.07	0.89	3.10	0.81
9C	4.40	4.66	4.35	3.91	3.29	3.60	2.00	3.87
10C	0.35	0.39	0.59	0.67	1.17	1.03	1.40	1.01
11C	2.29	0.73	0.84	1.38	0.68	0.94	0.17	1.18
12C	0.20	0.35	0.11	−0.30	0.16	−0.16	0.19	−0.25
13C	−0.07	2.79	2.31	1.38	2.95	2.34	3.65	1.80
14C	1.55	−1.32	−0.64	0.04	−1.04	−0.03	−0.91	0.34
15C	−5.21	−5.13	−4.89	−4.75	−3.75	−4.06	−3.67	−4.42
16C	−2.72	−3.24	−2.87	−2.88	−2.86	−2.95	−3.83	−3.07
17C	−3.56	−2.19	−2.28	−2.93	−1.38	−2.18	−0.98	−2.84

**Table 6:** Condensed dual descriptor $f^{\left(2\right)}{\left(r\right)}_{k}$ over the atoms of the trans-resveratrol molecule calculated with the M11, M11L, MN12L, MN12SX, N12, N12SX, SOGGA11 and SOGGA11X density functionals and the Def2TZVP basis set using water as a solvent simulated with the SMD parametrization of the IEF-PCM model. The actual values have been multiplied by 100 for an easier comparison. H atoms are not shown.

Atom	M11	M11L	MN12L	MN12SX	N12	N12SX	SOGGA11	SOGGA11X
1O	−0.08	−0.19	−0.17	−0.10	−0.23	−0.10	−0.18	−0.07
2O	−0.26	−0.23	−0.22	−0.22	−0.21	−0.21	−0.31	−0.20
3O	−4.63	−4.31	−4.65	−4.17	−4.75	−4.32	−5.44	−4.01
4C	4.39	3.30	3.57	3.63	2.93	3.55	2.93	4.01
5C	−6.55	−5.92	−5.65	−5.99	−4.86	−5.47	−5.34	−5.98
6C	0.63	0.74	0.21	0.33	0.23	0.02	0.73	0.04
7C	7.89	9.90	9.13	8.69	7.95	7.86	8.84	7.97
8C	2.48	1.00	1.17	1.72	1.17	1.65	1.76	1.83
9C	0.67	1.07	1.02	0.67	1.33	0.85	1.09	0.64
10C	1.81	1.25	1.33	1.54	1.20	1.50	1.43	1.72
11C	0.28	0.11	0.17	0.15	0.19	0.24	0.03	0.29
12C	−0.79	−0.52	−0.47	−0.83	−0.37	−0.67	−0.70	−0.86
13C	3.06	4.66	4.26	3.93	4.02	3.60	4.14	3.45
14C	−1.05	−3.92	−3.10	−2.38	−2.77	−2.10	−2.72	−1.92
15C	−2.72	−3.25	−3.03	−2.78	−2.78	−2.66	−2.92	−2.68
16C	−3.24	−2.88	−2.71	−2.85	−2.57	−2.68	−2.95	−2.83
17C	−2.12	−1.11	−1.13	−1.63	−0.71	−1.30	−0.70	−1.63

**Table 7:** Electrophilic $P_{k}^{+}$ and nucleophilic $P_{k}^{-}$ Parr functions over the atoms of the cis-resveratrol molecule calculated with the MN12SX and N12SX density functionals and the Def2TZVP basis set using water as a solvent simulated with the SMD parametrization of the IEF-PCM model. ASDs come from Hirshfeld and MBS population analysis. The actual values have been multiplied by 100 for an easier comparison. H atoms are not shown.

	MN12SX				N12SX			
	Hirshfeld		MBS		Hirshfeld		MBS	
Atom	$P_{k}^{+}$	$P_{k}^{-}$	$P_{k}^{+}$	$P_{k}^{-}$	$P_{k}^{+}$	$P_{k}^{-}$	$P_{k}^{+}$	$P_{k}^{-}$
1O	3.70	0.92	0.08	0.98	0.18	0.42	−0.06	0.50
2O	1.92	−0.49	−0.08	−0.45	−0.06	−0.72	−0.25	−0.64
3O	2.36	8.44	1.65	8.68	2.74	8.34	1.87	8.42
4C	9.42	3.85	9.81	1.55	8.88	3.41	8.70	0.31
5C	16.74	16.12	17.60	19.41	16.28	18.08	17.21	22.07
6C	4.80	13.04	1.68	16.95	4.33	12.52	0.43	16.24
7C	19.46	8.83	23.07	6.05	19.12	8.65	22.72	5.45
8C	6.50	8.45	7.15	11.05	7.09	8.49	8.27	11.44
9C	6.93	1.76	6.74	1.89	7.14	3.06	7.32	3.96
10C	2.15	1.22	−0.11	−0.71	1.63	0.23	−1.05	−2.59
11C	1.19	−0.38	−1.60	−4.04	0.44	−1.16	−2.92	−5.44
12C	6.26	4.22	7.24	2.64	6.84	4.33	8.43	3.10
13C	7.63	4.94	10.05	4.03	9.31	5.01	12.76	4.31
14C	8.60	10.20	11.73	13.51	9.39	11.56	13.03	15.71
15C	−0.16	5.27	−3.37	4.39	−1.15	4.27	−5.13	2.91
16C	−0.42	2.40	−4.33	0.57	−1.66	1.93	−6.62	−0.22
17C	7.98	11.22	12.68	13.51	9.50	11.60	15.29	14.48

**Table 8:** Electrophilic $P_{k}^{+}$ and nucleophilic $P_{k}^{-}$ Parr functions over the atoms of the trans-resveratrol molecule calculated with the MN12SX and N12SX density functionals and the Def2TZVP basis set using water as a solvent simulated with the SMD parametrization of the IEF-PCM model. ASDs come from Hirshfeld and MBS population analysis. The actual values have been multiplied by 100 for an easier comparison. H atoms are not shown.

	MN12SX				N12SX			
	Hirshfeld		MBS		Hirshfeld		MBS	
Atom	$P_{k}^{+}$	$P_{k}^{-}$	$P_{k}^{+}$	$P_{k}^{-}$	$P_{k}^{+}$	$P_{k}^{-}$	$P_{k}^{+}$	$P_{k}^{-}$
1O	0.37	−0.06	0.09	−0.01	0.16	−0.36	−0.07	−0.25
2O	−0.07	−0.28	−0.24	−0.22	−0.29	−0.64	−0.39	−0.54
3O	2.65	7.49	1.89	7.59	3.00	7.52	2.09	7.48
4C	8.06	3.43	7.23	0.09	7.66	2.86	6.44	−1.31
5C	15.57	17.01	16.01	20.54	15.11	17.73	10.31	21.68
6C	4.26	9.91	−0.28	11.69	3.52	9.60	−1.78	11.19
7C	20.27	9.81	24.09	8.05	20.14	9.54	24.17	7.58
8C	7.09	6.89	9.17	9.15	7.82	8.13	8.27	11.16
9C	6.44	5.66	6.54	7.63	6.91	6.44	7.43	9.00
10C	2.23	0.06	−0.05	−3.71	1.63	−0.84	−1.21	−5.44
11C	0.63	−0.17	−2.65	−4.23	−0.05	−1.15	−3.97	−6.15
12C	5.30	4.76	6.38	4.35	6.37	4.93	8.25	4.80
13C	10.23	5,47	14.37	5.36	11.27	5.77	16.16	5.99
14C	8.91	13.75	12.21	18.45	9.94	15.21	13.38	20.82
15C	0.27	3.15	3.42	1.52	−1.45	2.44	−5.52	0.39
16C	−0.38	2.42	−5.01	0.41	−1.80	1.62	−7.39	−0.86
17C	8.71	10.71	13.68	13.35	10.08	11.19	16.15	14.45

## Abbreviations

The following abbreviations are used in this manuscript:

DFT	Density Functional Theory
HOMO	Higher Occupied Molecular Orbital
LUMO	Lower Unoccupied Molecular Orbital
KID	Koopmans in DFT
IEF-PCM	Integral Equation Formalism-Polarized Continuum Model
SMD	Solvation Model Density
SCF	Self Consistent Field
ASD	Atomic Spin Density
MBS	Minimum Basis Set
